# Supplementary material for: Transpirational Leaf Cooling Effect Did Not Contribute Equally to Biomass Retention in Wheat Genotypes under High Temperature
Source: Plants (Basel). 2022 Aug 21;11(16):2174. doi: 10.3390/plants11162174 (PMC9416376; doi:10.3390/plants11162174)
Supplement: Supplementary file 1 [file plants-11-02174-s001.zip › plants-1830076-supplementary.pdf]

plants

Article

# Transpirational Leaf Cooling Effect Did Not Contribute Equally to Biomass Retention in Wheat Genotypes under High Temperature

SUPPLEMENTARY FIGURES

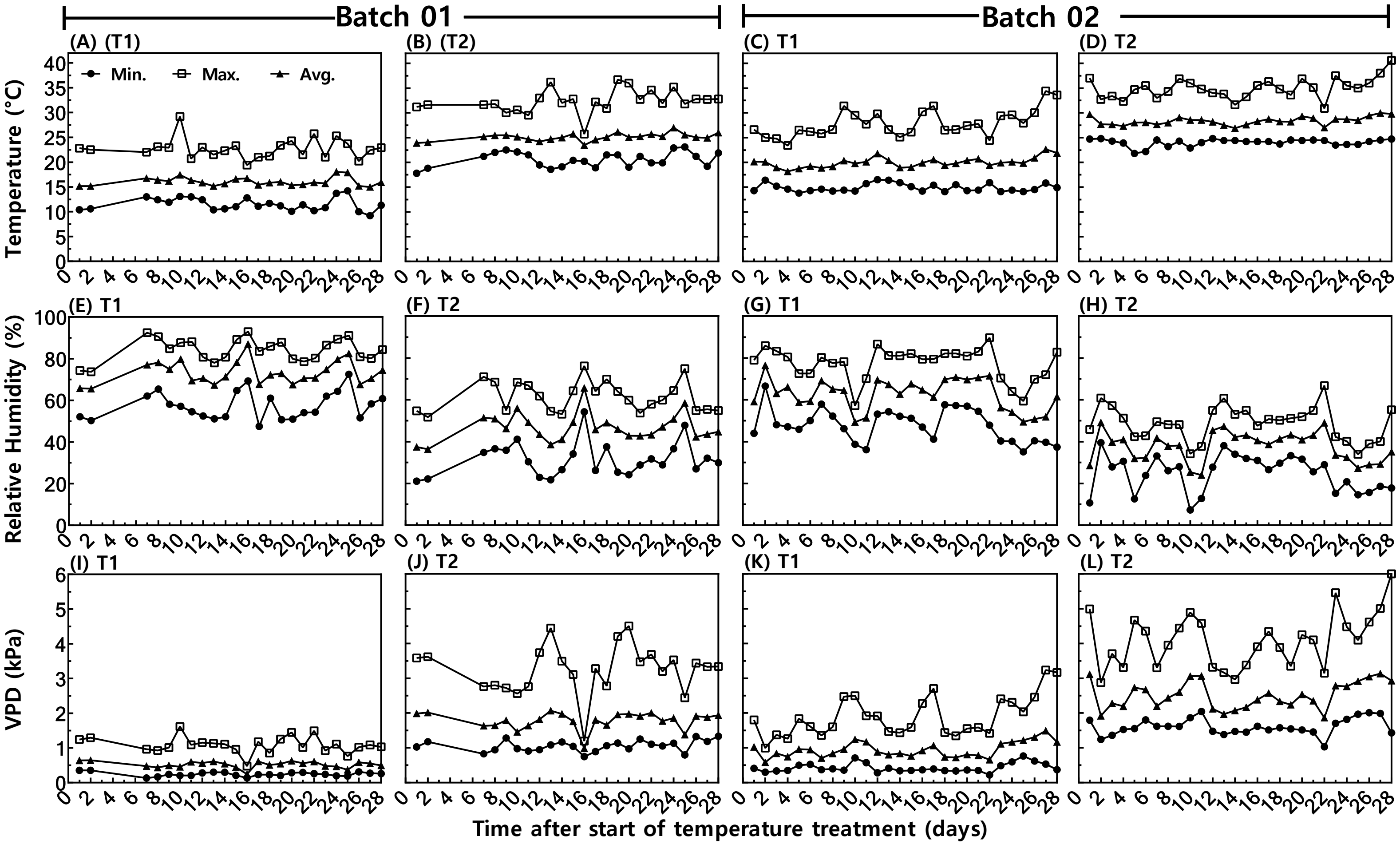

Figure S1. Daily fluctuations in maximum, minimum, and average environmental conditions inside the two glasshouses during the experimental period (after imposing the temperature treatment). Plants were grown in the ambient temperature (Amb-T1) glasshouse and half of the replicates transferred to the high temperature (High-T2) glasshouse 7 weeks after planting.

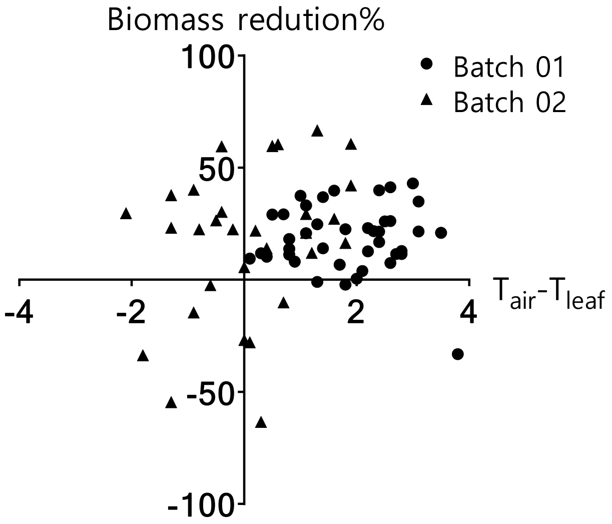

Figure S2. Relationship between biomass reduction% and leaf-to-air temperature differential (T_{air}–T_{leaf}) in Batch 01 and Batch 02 wheat genotypes under T2 temperature condition. Biomass reduction% refers to the difference in biomass between T1 and T2 temperature conditions in relation to the biomass under T1 condition. Each data point represents an individual plant, all genotypes combined.

SUPPLEMENTARY TABLES

Table S1. Environmental conditions inside the two glasshouses during the experiment period (after imposing the temperature treatment). Plants were grown in the ambient temperature (Amb-T1) glasshouse and half of the replicates transferred to the high temperature (High-T2) glasshouse 7 weeks after planting. Data are means ± SEM of the minimum (night), maximum (day) and average conditions of the particular parameter measured.

	Glasshouse temperature (°C)			Relative humidity (%)			Vapour pressure deficit (kPa)		
	**Min.**	**Max.**	**Avg.**	**Min.**	**Max.**	**Avg.**	**Min.**	**Max.**	**Avg.**
**Batch 01**									
Amb (T1)	11.5 ± 0.3	22.7 ± 0.4	16.1 ± 0.2	57.4 ± 1.4	84.2 ± 1.1	73.2 ± 1.1	0.24 ± 0.01	1.09 ± 0.05	0.52 ± 0.02
High (T2)	20.8 ± 0.3	32.3 ± 0.5	25.1 ± 0.1	31.6 ± 1.7	61.9 ± 1.5	46.8 ± 1.4	1.06 ± 0.03	3.25 ± 0.14	1.78 ± 0.05
**Batch 02**									
Amb (T1)	14.9 ± 0.1	27.9 ± 0.5	20.0 ± 0.2	47.8 ± 1.5	77.3 ± 1.5	62.6 ± 1.4	0.43 ± 0.02	1.89 ± 0.11	0.94 ± 0.04
High (T2)	24.0 ± 0.1	35.0 ± 0.4	28.4 ± 0.1	24.8 ± 1.6	48.8 ± 1.5	37.5 ± 1.3	1.61 ± 0.04	4.09 ± 0.15	2.52 ± 0.07

plants

Table S2. Parameters of linear regression models for the relationship between instantaneous rate of transpiration (T_r) and atmospheric vapour pressure deficit (VPD) in 20 wheat genotypes. Data are means ± SEM for the best-fit values and the goodness of fits of the regressions.

Genotype	Slope (mmol H_2O m^{-2} s^{-1} kPa^{-1})	Instantaneous rate of T_r-intercept (mmol H_2O m^{-2} s^{-1})	R^2	P
Hartog	1.04 ± 0.39	5.50 ± 1.14	0.22	0.015
Kukri	1.06 ± 0.34	4.98 ± 0.95	0.31	0.005
Drysdale	1.14 ± 0.47	6.11 ± 1.27	0.22	0.025
Espada	1.22 ± 0.31	4.38 ± 0.85	0.42	0.001
Sonora 64	1.53 ± 0.22	1.65 ± 0.62	0.72	< 0.0001
Mace	1.57 ± 0.29	5.17 ± 0.83	0.56	< 0.0001
Downey	1.59 ± 0.24	0.46 ± 0.73	0.71	< 0.0001
Gladius	1.66 ± 0.34	4.19 ± 0.96	0.51	< 0.0001
RAC 875	1.67 ± 0.34	4.64 ± 1.00	0.49	< 0.0001
Glossy-Huguenot	1.69 ± 0.41	5.09 ± 1.24	0.42	0.001
Ciano 67	1.71 ± 0.46	1.91 ± 1.35	0.41	0.001
Emu Rock	1.75 ± 0.24	4.24 ± 0.65	0.72	< 0.0001
Wyalkatchem (Batch 01)	1.87 ± 0.28	1.85 ± 0.78	0.69	< 0.0001
Wyalkatchem (Batch 02)	1.56 ± 0.29	5.00 ± 0.82	0.55	< 0.0001
Yecora 70	1.93 ± 0.44	1.65 ± 1.32	0.49	0.0003
Janz	2.24 ± 0.53	0.62 ± 1.64	0.51	0.001
LongReach-Envoy	2.46 ± 0.48	2.37 ± 1.41	0.52	< 0.0001
Glennson 81	2.85 ± 0.35	0.37 ± 1.03	0.77	< 0.0001
Excalibur	3.05 ± 0.48	0.40 ± 1.40	0.67	< 0.0001
Einkorn	3.05 ± 0.38	0.70 ± 1.15	0.78	< 0.0001
Magenta	3.08 ± 0.42	-0.45 ± 1.22	0.72	< 0.0001
